# Efficacy and Safety of Vonoprazan in Patients With Nonerosive Gastroesophageal Reflux Disease: A Randomized, Placebo-Controlled, Phase 3 Study

**DOI:** 10.14309/ctg.0000000000000101

**Published:** 2019-11-02

**Authors:** Yoshikazu Kinoshita, Yuuichi Sakurai, Nobuyoshi Takabayashi, Kentaro Kudou, Takahiro Araki, Takuya Miyagi, Katsuhiko Iwakiri, Kiyoshi Ashida

**Affiliations:** 1 Department of Gastroenterology and Hepatology, Shimane University School of Medicine, Izumo, Japan;; 2 Takeda Development Center Japan, Takeda Pharmaceutical Company Limited, Osaka, Japan;; 3 Department of Internal Medicine, Fukuyama Daiichi Hospital, Hiroshima, Japan;; 4 Department of Gastroenterology, Nippon Medical School Graduate School of Medicine, Tokyo, Japan;; 5 Department of Gastroenterology, Rakuwakai Otowa Hospital, Kyoto, Japan.

## Abstract

**OBJECTIVES:**

To assess the efficacy and safety of vonoprazan on heartburn symptoms in patients with nonerosive reflux disease (NERD) (ClinicalTrials.gov: NCT02954848).

**METHODS:**

This phase 3, double-blind, placebo-controlled study included Japanese patients aged 20 years and older with grade N/M NERD and recurrent heartburn. Patients received placebo (n = 245) or vonoprazan 10 mg (n = 238) for 4 weeks. The primary efficacy outcome was frequency of heartburn experienced by patients during the treatment period (proportion of days without heartburn). Other outcomes included cumulative improvement rates of heartburn, proportion of patients with complete heartburn resolution in the fourth week of treatment, and safety.

**RESULTS:**

Compared with placebo, the proportion of days without heartburn was not significantly higher in the vonoprazan group in the full analysis (primary end point, 72.55% vs 61.50%, vonoprazan vs placebo, *P* = 0.0643) but was significantly higher in the per-protocol-set sensitivity analysis (*P* = 0.0341). Early onset of response and significantly greater cumulative improvement rates of heartburn were observed in the vonoprazan group (*P* = 0.0003). In a post hoc analysis, a greater proportion of patients with complete heartburn resolution in the fourth week of treatment were reported in the vonoprazan group (*P* = 0.0023). Incidence of treatment-emergent adverse events was similar between treatment groups (23.5% vs 23.3%); most treatment-emergent adverse events were mild in severity.

**DISCUSSION:**

Although vonoprazan 10 mg was not superior to placebo with respect to proportion of days without heartburn in Japanese patients with NERD, vonoprazan had a significantly higher cumulative rate of heartburn resolution and was well tolerated.

## INTRODUCTION

Nonerosive reflux disease (NERD) is common in Western and Asian countries such as Japan and is associated with a significant burden on patient life and on health care systems (1–5). The clinically relevant goal for NERD treatment is to relieve acid reflux symptoms (i.e., heartburn), and the first-line treatment is proton pump inhibitors (PPIs) (6). Despite their well-documented clinical efficacy and safety, PPIs display several limitations, one of which is that only ∼50%–60% of patients with NERD achieve symptom resolution by 4 weeks (7). Therefore, there is an unmet need for novel drugs to improve outcomes in patients with NERD.

Unlike conventional PPIs, vonoprazan fumarate (Takecab or Vocinti, Takeda, Osaka, Japan) competitively inhibits H+, K+ -ATPase by binding to the potassium ion binding site and does not require activation by gastric acid (8). Vonoprazan has been approved in Japan for the treatment of gastric and duodenal ulcers, healing of reflux esophagitis and prevention of relapse, secondary prevention of low-dose aspirin– or nonsteroidal anti-inflammatory drug–induced gastric mucosal damage, and first- and second-line *Helicobacter pylori* eradication therapy (9). For NERD, a previous randomized controlled study in Japanese patients did not demonstrate the superiority of vonoprazan 10 mg to placebo (10). Several reasons for this have been posited, including the symptom scale for assessing heartburn used in the study and inappropriate patient selection (i.e, inclusion of patients with heartburn unrelated to acid reflux, such as functional dyspepsia or functional heartburn).

To address these questions, we have conducted this double-blind, randomized, placebo-controlled trial in Japanese patients with NERD. The study objectives were to assess the safety and efficacy of vonoprazan 10 mg, compared with placebo, on heartburn symptoms (assessed by proportion of days without heartburn and cumulative improvement rates of heartburn) during 4 weeks of treatment.

## METHODS

### Study design

This was a multicenter, randomized, double-blind, placebo-controlled, phase 3 trial of oral once-daily vonoprazan 10 mg compared with placebo in adults with NERD. The study was registered (ClinicalTrials.gov: NCT02954848), approved by the local institutional review board, and conducted at 44 study sites in Japan (November 2016–February 2018) in accordance with the Declaration of Helsinki, the International Conference on Harmonisation Guideline for Good Clinical Practice, and applicable Japanese regulations. All patients signed the informed consent form before participation.

### Study population

Included patients were aged 20 years and older, with an endoscopic diagnosis of NERD grade N (normal mucosa) or M (minimal changes to the mucosa, e.g., erythema and/or whitish turbidity) (Modified Los Angeles Classification) (11) and recurrent heartburn (≥2 d/wk for the past 3 weeks), and if, during the run-in period, they had demonstrated compliance (≥75%) with study drug administration, had heartburn (≥2 days), and had completed all required information in the patient diary. Recurrent heartburn was defined as a burning sensation arising from the stomach or behind the breastbone, which commonly appeared/was exacerbated postprandially or when bending forward or during abdominal compression. The endoscopic severity of NERD was assessed by the investigators and was confirmed by a Central Adjudication Committee.

Patients were excluded if they had a history of suspected functional upper gastrointestinal disorders (e.g., functional dyspepsia, diagnosed by the Rome IV criteria), long-segment Barrett's esophagus (≥3 cm), any upper gastrointestinal surgery or surgery that may affect gastroesophageal reflux, acute gastritis, acute esophageal bleeding or gastric/duodenal ulcers, Zollinger-Ellison syndrome, or concurrent upper gastrointestinal symptoms more severe than heartburn.

Full inclusion/exclusion criteria are available at www.clinicaltrials.gov (12).

All concomitant medications, including vitamin supplements, over-the-counter drugs, and herbal medicines, were recorded. Excluded medications were PPIs, H_2_-receptor antagonists, prokinetics, anticholinergics, prostaglandins, antacids, antigastrin drugs, mucosal protective agents, triple therapy for *H. pylori* eradication, atazanavir sulfate, rilpivirine hydrochloride, antidepressants, anxiolytics, and investigational or postmarketing study products.

### Interventions

In the run-in period, patients underwent upper gastrointestinal endoscopy and received 1 week of placebo treatment in a single blinded manner. Subsequently, eligible patients were randomized 1:1 (stratified according to their grade N or M) to receive 1 tablet of vonoprazan 10 mg or placebo orally once daily after breakfast for 4 weeks. Patients were followed up at weeks 2 and 4. Treatment compliance was assessed using patient diaries and examination of the study drug container. The patient was to be withdrawn from the study if the compliance rate was <50% for 2 successive visits.

### Sample size

The sample size was calculated using the patient numbers required for efficacy evaluations in each grade group and the proportion of days without heartburn determined in previous studies of lansoprazole (mean ± SD: lansoprazole 15 mg 63.2% ± 32.20% vs placebo 51.0% ± 28.39%) and vonoprazan (vonoprazan 10 mg 28.9% ± 34.85% vs placebo 22.6% ± 28.20%) (10,13). Enrollment of patients with either grade N or M ended when the number of enrolled patients with each grade exceeded 332 or 70% of the total planned number of patients. Assuming that the difference between treatment groups would be 10% with a common SD of 32%, 230 patients per treatment group were required to ensure 90% power of the 2-sample Wilcoxon rank-sum test to verify the intergroup difference with a significance level of 0.05. Assuming a 3% dropout, 237 patients per treatment group were required.

### Randomization

Eligible patients were randomized to the study drugs according to the medication identification number allocated to each study site. The sponsor randomization personnel or designee generated the randomization schedule using block randomization. All randomization information was stored in a secured area and accessible only by authorized personnel.

### Blinding

Vonoprazan and placebo tablets were indistinguishable from each other. Patients, investigators, and all study personnel remained blinded to the randomized treatment assignments until the database lock. Emergency code keys were kept by the Emergency Key Control Center and only broken during an emergency or after database lock. Serum gastrin and pepsinogen I/II levels were not disclosed during the data collection period.

### Efficacy outcome measures

Primary efficacy outcome was the frequency of heartburn experienced by patients during the 4-week treatment period as measured by the proportion of days without heartburn. The patients recorded their daily heartburn symptoms from waking to bedtime and their compliance with the study treatment.

Secondary outcomes were the cumulative improvement rates of heartburn, and the proportion of days without heartburn in patient subgroups as stratified by response to treatment (improved, not improved) at week 2, baseline endoscopic findings (grade N/M), or a combination of endoscopic findings and response at week 2. Improvement at week 2 was defined as heartburn on <2 of the 7 days before week 2 (criterion 1) or a lower proportion of days with heartburn up to week 2 than during the run-in period (criterion 2). An independent central evaluation committee reviewed all endoscopic assessments reported by the sites in a consistent manner. In addition, the proportion of patients without heartburn or no hindrance to daily activities in the fourth week of the treatment period was assessed in a post hoc analysis.

### Safety outcome measures

The type and frequency of treatment-emergent adverse events (TEAEs; coded using the Medical Dictionary for Regulatory Activities version 20.0 for analysis) and serious adverse events were assessed continuously. Serum chemistry, hematology, and urinalysis findings, serum gastrin and pepsinogen I/II concentrations, and vital signs and electrocardiogram results were recorded at each study visit.

### Statistical analysis

Three analysis sets were defined in this study: the full analysis set (FAS), per-protocol set (PPS), and safety analysis set. The FAS and safety analysis set included all randomized patients who received ≥1 dose of study drug. The PPS included all patients in the FAS who had available measurements for the primary variable, a prespecified minimal exposure to the treatment regimen, and no major protocol violations.

The primary and secondary efficacy end points were analyzed in the FAS. Descriptive statistics were used to summarize the proportion of days without heartburn during the treatment period by treatment group. A Hodges-Lehmann estimation was used to estimate the median difference between treatment groups, and a 2-sample Wilcoxon rank-sum test was used for comparison between treatment groups. The Kaplan-Meier method was used to estimate the cumulative improvement rates of heartburn during the treatment period for each treatment group. The event date was defined as the first confirmed date of heartburn improvement that continued until the last day of study treatment. The censoring date was defined as 6 days before the last day with documentation of whether the patients experienced heartburn (applicable only to patients without symptom improvement). A log-rank test was used to compare the cumulative improvement rates of heartburn between treatment groups. The frequency of the proportion of days without heartburn for each treatment group was presented in a histogram. A sensitivity analysis for primary end points was performed in the PPS to confirm the robustness of the analysis.

For all analyses, 2-sided 95% confidence intervals for median between-group differences were calculated.

For the proportion of patients without heartburn or no hindrance to daily activities in the fourth week of the treatment period, the point estimate and 2-sided 95% confidence interval of the percentage difference were calculated between treatment groups, and a Fisher exact test was used for the comparison between treatment groups.

For the safety end points, continuous variables (observed values and changes from study drug start) were summarized by treatment group for each visit using descriptive statistics. For categorical variables, shift tables in each category before and after treatment for the treatment period postbaseline visit were provided for each treatment group.

All available efficacy and safety data were included in data listing and tabulations. No imputation of values for missing data was performed unless otherwise specified. For clinical laboratory tests, values less than the quantification's lower limit were treated as zero when calculating the descriptive statistics.

The same analyses were performed in the subgroups. Analyses were conducted using SAS version 9.4 (SAS Institute Inc, Cary, NC).

## RESULTS

### Patient disposition, demographic, and baseline clinical characteristics

**Figure 1.**
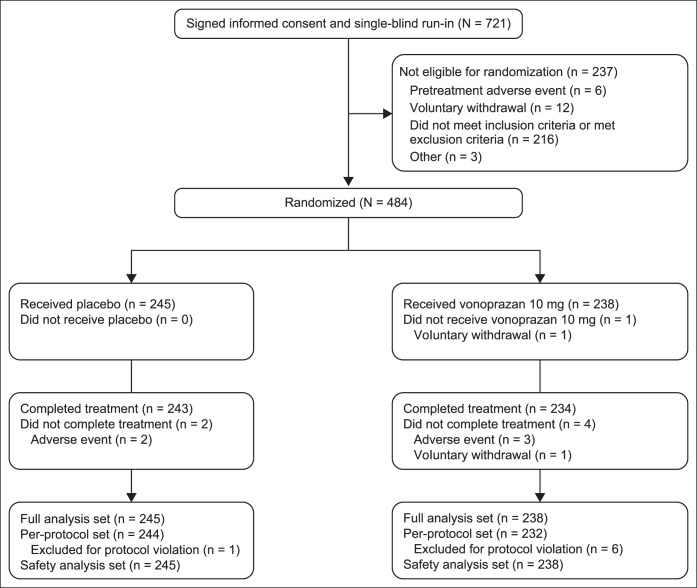
Patient disposition. The full analysis and safety analysis sets were defined as all patients who were randomized and received at least 1 dose of the study drug. The per-protocol set was defined as all patients in the full analysis set who had available measurements for the primary variable, a prespecified minimal exposure to the treatment regimen, and no major protocol violations.

Of 721 patients who gave informed consent, 484 were randomized for treatment; however, 1 patient in the vonoprazan group did not receive the study drug and was excluded from the FAS (placebo 245; vonoprazan 238) (Figure 1). No obvious differences were observed between treatment groups at baseline (Table 1).

**Table 1.**
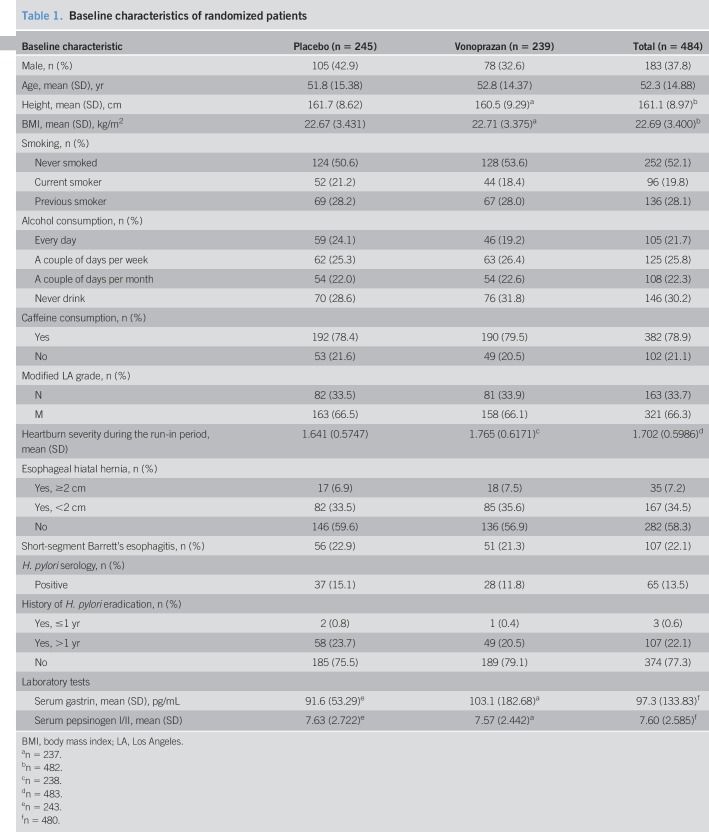
Baseline characteristics of randomized patients

### Efficacy outcome measures

**Figure 2.**
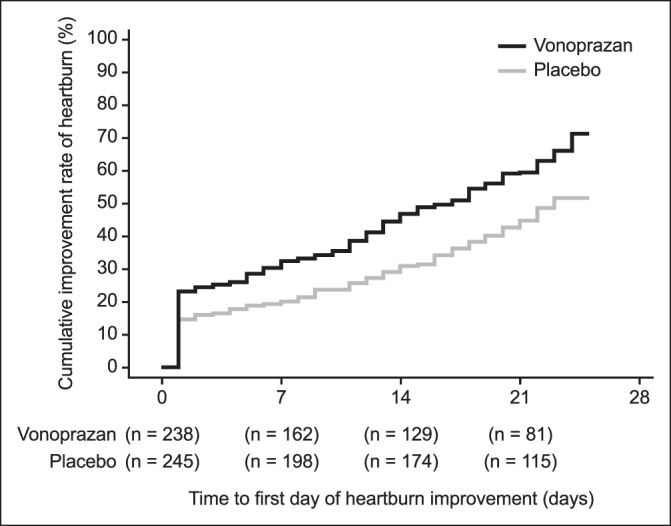
Cumulative improvement rate of heartburn during the treatment period in the full analysis set.

**Figure 3.**
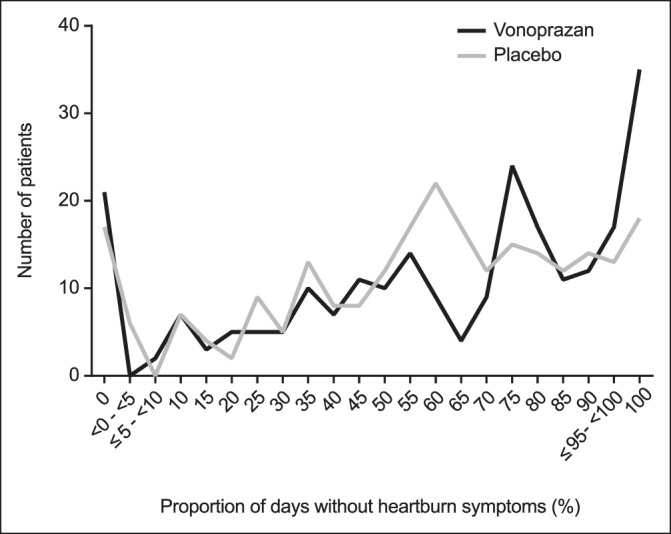
Histogram presenting proportion of days without symptoms of heartburn during the treatment period in the full analysis set.

Compared with placebo, treatment with vonoprazan improved the proportion of days without symptoms of heartburn during the treatment period and significantly increased cumulative improvement rates of heartburn. Although the proportion of days without heartburn during the treatment period was not statistically different between treatment groups in the FAS (primary end point, *P* = 0.0643), this was significantly higher with vonoprazan than with placebo (*P* = 0.0341) in the sensitivity analysis comprising the PPS population (Table 2). Differences in the cumulative improvement rates of heartburn during the treatment period between treatment groups were statistically significant (*P* = 0.0003, log-rank test) (Figure 2). The difference was observed after 1 day of treatment and maintained throughout the treatment period. In addition, the distribution pattern indicated a shift in the vonoprazan group to a higher proportion of days without heartburn during the treatment period compared with the placebo group (Figure 3). In a post hoc analysis, the proportion of patients without heartburn or no hindrance to daily activities in the fourth week of the treatment period was significantly higher with vonoprazan than with placebo (*P* = 0.0023) (see Table, Supplementary Digital Content 1, http://links.lww.com/CTG/A115).

**Table 2.**
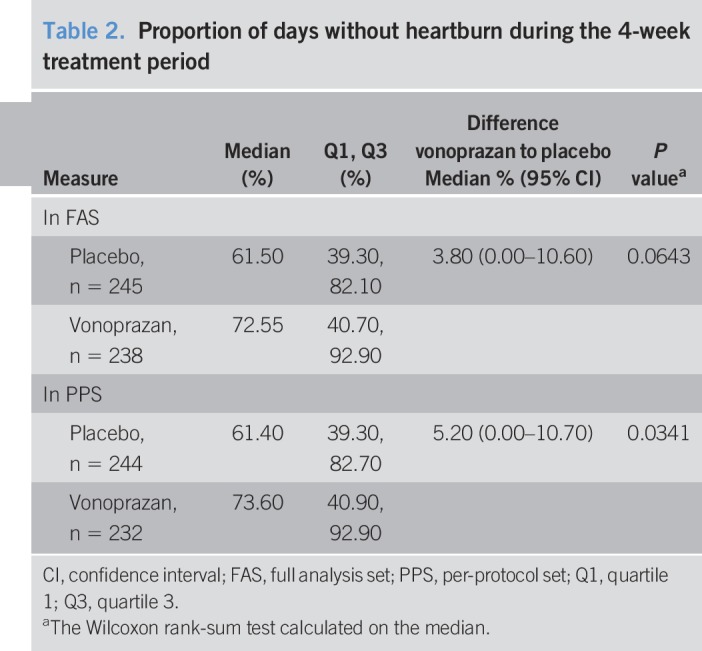
Proportion of days without heartburn during the 4-week treatment period

#### Outcome measures in the subgroup analyses

The improvement in heartburn symptoms by week 2 was associated with a greater proportion of days without heartburn during the treatment period in the vonoprazan group compared with the placebo group (*P* < 0.05), regardless of the improvement criteria used (see Table, Supplementary Digital Content 2, http://links.lww.com/CTG/A115).

By contrast, no differences between treatment groups were noted when patients were stratified by endoscopic grade (see Table, Supplementary Digital Content 2, http://links.lww.com/CTG/A115).

In patients with grade M, improvement in heartburn symptoms by week 2 (criterion 1) was associated with a greater proportion of days without heartburn during the treatment period in the vonoprazan group compared with the placebo group (*P* < 0.05); however, this was not observed for patients with improvement defined by criterion 2 (see Table, Supplementary Digital Content 2, http://links.lww.com/CTG/A115). In patients with grade N, improvement in heartburn symptoms using criterion 2 was associated with significantly greater treatment response for vonoprazan compared with placebo.

### Safety outcome measures

Over the treatment period, the overall incidences of TEAEs, drug-related TEAEs, and TEAEs leading to discontinuation were similar between the groups (Table 3). Most TEAEs were mild in intensity. One serious adverse event was recorded (colitis ulcerative, placebo group, occurred during the treatment period, unrelated to the study drug); no deaths were reported. Viral upper respiratory tract infection was the only TEAE reported with an incidence ≥2%; however, it was considered unrelated to the study drug. Mean levels of serum gastrin, pepsinogen I, and pepsinogen II increased after study drug administration in the vonoprazan group, while no clinically significant changes were observed in the placebo group (Table 4).

**Table 3.**
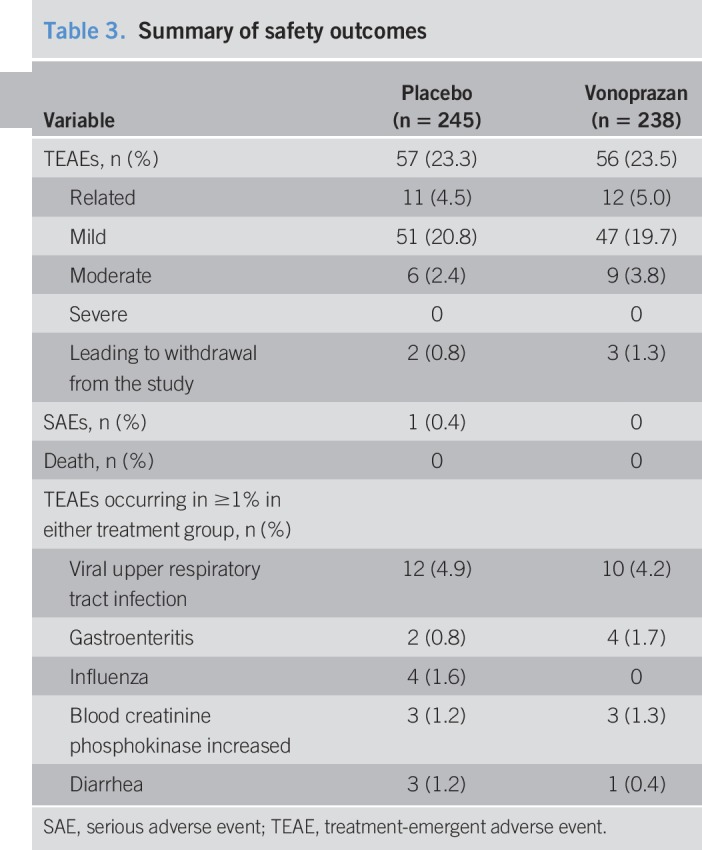
Summary of safety outcomes

**Table 4.**
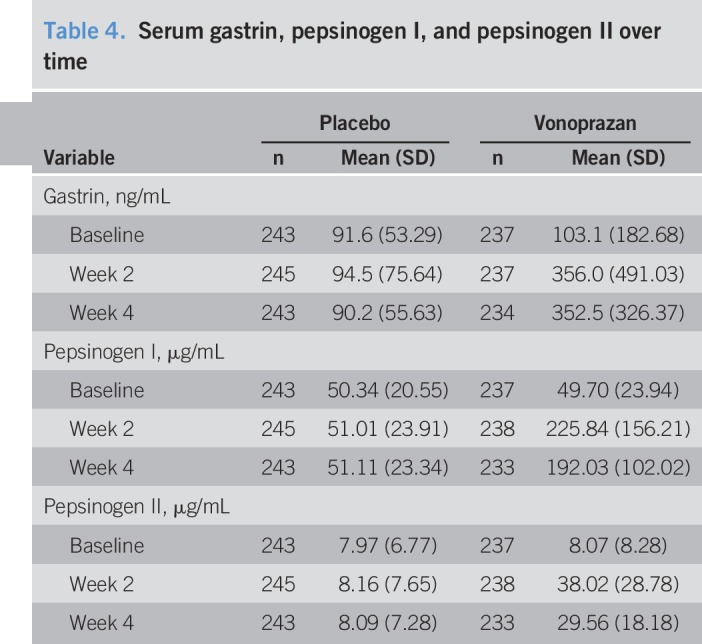
Serum gastrin, pepsinogen I, and pepsinogen II over time

## DISCUSSION

This randomized, placebo-controlled trial has confirmed the efficacy and safety of vonoprazan 10 mg in patients with NERD. Although the results showed that vonoprazan was not superior to placebo in the FAS with respect to the proportion of days without heartburn, the superiority of vonoprazan to placebo was indicated in the PPS analysis. Furthermore, the study showed the rapid and sustained heartburn relief of vonoprazan compared with placebo. The safety profile of vonoprazan was comparable with placebo; most TEAEs were mild in severity.

The study findings confirm and extend the results of the recent phase 3 trial reported by Kinoshita et al. (10). However, several differences between these 2 studies should be mentioned. Our study showed that vonoprazan 10 mg was superior to placebo in the PPS population, whereas in the previous study, vonoprazan 10 mg was not superior to placebo in the FAS or the PPS population (Kinoshita et al., unpublished data, 2016). Moreover, we found that vonoprazan significantly increased the cumulative improvement rates of heartburn during the treatment period compared with placebo, in contrast with the previous study. In our study, the cumulative improvement rate for vonoprazan was about 30% at 7 days, while in the previous study the cumulative improvement rate at 7 days was <10%. Looking at an even earlier time point (day 1), the improvement rate with vonoprazan in our study was already >20%. This more favorable response may be explained by the reduction of confounding factors in the previous study, such as the inclusion of patients with functional disorders, placebo responders, or the exclusion of patients who responded to antacids (10). Notably, in the current study, despite trying to exclude placebo responders, a higher proportion of days without heartburn and cumulative improvement rates of heartburn was observed in both vonoprazan and placebo groups, as expected in clinical trials with gastroesophageal reflux disease (14). Importantly, similar response rates were recorded in 2 retrospective studies conducted in Japan, where vonoprazan improved gastroesophageal reflux symptoms (symptomatic improvement rates were about 69% in patients with NERD) (15,16).

Compared with the results from studies of rabeprazole or omeprazole, our study showed comparable results for the reduction of heartburn symptoms in the fourth week or the last week of the treatment period in patients with NERD (17,18). Patients receiving rabeprazole 10 mg had a significantly higher rate of complete resolution of heartburn compared with placebo in the fourth week or the last week of the 4-week treatment (44% vs 21%, *P* = 0.001) (17). Similarly, the difference vs placebo in the rate of complete resolution of heartburn during week 4 with omeprazole 10 mg was 20% (*P* < 0.001), which is consistent with the rate of 14% (*P* = 0.0023) seen in our study (18). However, there were a number of patients who did not respond to vonoprazan (proportion of days without heartburn symptoms 0%) (Figure 3), suggesting that heartburn might not have been caused by acid reflux in these patients. Functional heartburn and esophageal dysmotility, which are known to be NERD-like symptoms that respond poorly to acid suppressants, occur in Japanese patients with NERD (19); hence, it is possible that these patients were not completely excluded from our study.

Response to vonoprazan treatment at 2 weeks may predict response at 4 weeks, which was similar to the findings reported in Kinoshita et al. (10). Baseline endoscopic grade was not predictive of response at 4 weeks; these findings are consistent with those of other studies conducted in Japan with vonoprazan 10 mg (10) or omeprazole (18). Thus, response to treatment at 2 weeks may be useful in deciding whether to continue the course of treatment or identify an alternate therapy.

It remains unclear whether the response to acid-reducing treatments such as vonoprazan or conventional PPIs differs between grade M or N NERD. Results from our study showed that although there was no significant difference between treatment groups for the proportions of days without heartburn, there were similar proportions of days without heartburn observed in patients who were receiving vonoprazan with different grades of NERD (grade M 67.90%, grade N 76.70%, see Table, Supplementary Digital Content 2, http://links.lww.com/CTG/A115). Similarly, a previous study reported no significant difference between vonoprazan 10 mg and placebo for the proportions of days without heartburn in patients with grade M or N NERD (10). The results for vonoprazan are similar to those reported for omeprazole (18). These data suggest that the categories of grade M or grade N may not be relevant with reducing heartburn symptoms.

The safety data reported in the current study are consistent with those of other studies with vonoprazan (10,20–22). Most TEAEs were classified as mild in intensity. Consistent with the mechanism of action of vonoprazan, and similar to PPIs, higher rates of gastroenteritis and increased serum gastrin, pepsinogen I, and pepsinogen II levels were observed after vonoprazan administration (10,23–26).

Strengths of the study include its prospective, randomized, controlled design with a run-in period that could reduce the known placebo effect observed in studies with PPIs for gastroesophageal reflux disease treatment (14); the absence of antacid use during the run-in period that reflects clinical practice in NERD treatment and avoids excluding antacid responders; the use of a vonoprazan dose and duration that reflect clinical practice for NERD in Japan; and the inclusion of patients with endoscopically confirmed NERD. Finally, a study-specific committee comprising independent experts in endoscopic grading of NERD ensured that the endoscopic diagnosis was confirmed in a consistent manner. Limitations of the study include its non–active-controlled design, the lack of a validated patient-reported outcome instrument for heartburn symptoms, and the generalizability of the study to non-Japanese populations. The placebo-controlled design was selected for study feasibility because large variability in the evaluation of NERD would make the sensitivity of this study unreliable if conducted with an active-controlled noninferiority design. Since NERD is influenced by diet, lifestyle, and obesity (27), the effect of vonoprazan in other populations will need to be investigated.

In conclusion, although the FAS analysis showed that vonoprazan 10 mg was not superior to placebo with respect to the proportion of days without heartburn in patients with NERD, the PPS sensitivity analysis showed a significantly greater improvement in the vonoprazan group compared with placebo on this end point. In addition, vonoprazan delivered rapid relief from heartburn symptoms, had a significantly higher cumulative rate of heartburn resolution, and was well tolerated. The partial response of vonoprazan monotherapy in this study may be explained by the heterogeneous pathophysiology of NERD.

## CONFLICTS OF INTEREST

**Guarantor of the article:** Yoshikazu Kinoshita, MD, PhD.

**Specific author contributions:** All authors participated in the interpretation of study results and in the drafting, critical revision, and approval of the final version of the manuscript. Y.K., Y.S., N.T., K.K., and T.A. were involved in the study design. K.K. conducted the statistical analyses. Y.K. was a coordinating investigator, and T.M. was the study medical expert. K.I. and Y.A. were members of the Central Adjudication Committee for endoscopic grading of NERD.

**Financial support:** This study was sponsored by Takeda Pharmaceutical Company Limited, manufacturer/licensee of Takecab or Vocinti. Takeda Pharmaceutical Company Limited was involved in the study design, data collection, data analysis, and preparation of the manuscript. Medical writing assistance was provided by Thao Le, MD, PhD, and Tania Dickson, PhD, CMPP, of ProScribe—Envision Pharma Group and was funded by Takeda Pharmaceutical Company Limited. ProScribe's services complied with international guidelines for Good Publication Practice (GPP3). Ethical approval and informed consent: The study was registered (ClinicalTrials.gov: NCT02954848), approved by the local institutional review board, and conducted at 44 study sites in Japan in accordance with the Declaration of Helsinki, the International Conference on Harmonisation Guideline for Good Clinical Practice, and applicable Japanese regulations. All patients signed the informed consent form before participation.

**Potential competing interests:** Y.K. has received research funding, honoraria, and other remunerations from Abbott, Ajinomoto, Astellas Pharma, AstraZeneca, Daiichi Sankyo, EA Pharma, Eisai, JIMRO, Mylan EPD, Otsuka Pharmaceutical, Scanpo Pharma, Takeda Pharmaceutical Company Limited, and Zeria Pharmaceutical. Y.S., N.T., K.K., and T.A. are employees of Takeda Pharmaceutical Company Limited. T.M. has no conflicts of interest to declare. K.I. and K.A. have received honoraria and other remunerations from Otsuka Pharmaceutical and Takeda Pharmaceutical Company Limited.

**Study Highlights**

**WHAT IS KNOWN**

✓ Novel drugs to improve outcomes in patients with NERD are required.

**WHAT IS NEW HERE**

✓ In patients with NERD and recurrent heartburn, vonoprazan 10 mg was not superior to placebo for the proportion of days without heartburn.
✓ Sensitivity analyses showed that vonoprazan significantly reduced the proportion of days without heartburn compared with placebo.
✓ Compared with placebo, vonoprazan induced an early response and a significantly greater cumulative heartburn improvement rate.
✓ Improvement in heartburn symptoms at 2 weeks was associated with a significantly better response to vonoprazan at 4 weeks.
✓ Vonoprazan was well tolerated, with a safety profile similar to placebo.

**TRANSLATIONAL IMPACT**

✓ The results from this study may be useful for further understanding NERD.

## Supplementary Material

SUPPLEMENTARY MATERIAL

## APPENDIX

### Off-label disclosure

Vonoprazan (Takecab or Vocinti) was approved in Japan in 2015 for erosive esophagitis, gastric ulcer, duodenal ulcer, prevention of recurrent gastric or duodenal ulcer during low-dose aspirin therapy, prevention of recurrent gastric or duodenal ulcer during nonsteroidal anti-inflammatory drug therapy, and adjunct to *H. pylori* eradication. Use of vonoprazan for the treatment of nonerosive reflux disease is not labeled in Japan. Vonoprazan is not approved by the US Food and Drug Administration or the European Medicines Agency.

### Data sharing

Takeda Pharmaceutical Company Limited makes patient-level, deidentified data sets and associated documents available after applicable marketing approvals and commercial availability have been received, an opportunity for the primary publication of the research has been allowed, and other criteria have been met as set forth in Takeda's Data Sharing Policy (see https://www.takedaclinicaltrials.com/for details). To obtain access, researchers must submit a legitimate academic research proposal for adjudication by an independent review panel, who will review the scientific merit of the research and the requestor's qualifications and conflict of interest that can result in potential bias. Once approved, qualified researchers who sign a data sharing agreement are provided access to these data in a secure research environment.
