# Supplementary material for: Efficacy and Safety of Vonoprazan in Patients With Nonerosive Gastroesophageal Reflux Disease: A Randomized, Placebo-Controlled, Phase 3 Study
Source: Clin Transl Gastroenterol. 2019 Nov 2;10(11):e00101. doi: 10.14309/ctg.0000000000000101 (PMC6890278; doi:10.14309/ctg.0000000000000101)
Supplement: SUPPLEMENTARY MATERIAL [file ct9-10-e00101-s001.docx]

SUPPLEMENTAl digital content

**Table, Supplemental Digital Content 1. Estimate of proportion of patients with no symptoms of heartburn or no hindrance to daily activities in the fourth week of the treatment period**

| **Treatment** | **Estimate**  **% (95% CI)** | **Difference  vonoprazan to placebo**  **% (95% CI)** | ***P* value^a^** |
| --- | --- | --- | --- |
| Placebo, n = 245 | 35.5 (29.5, 41.5) | 14.1 (5.3, 22.8) | 0.0023 |
| Vonoprazan, n = 238 | 49.6 (43.2, 55.9) |  |  |

CI, confidence interval.

^a^Fisher’s exact test.

**Table, Supplemental Digital Content 2.** **Subgroup analyses of proportion of days without heartburn by response to treatment at Week 2, baseline NERD grade, or combination of baseline NERD grade and response to treatment at Week 2**

|  |  | **Placebo** |  | **Vonoprazan** | ***P* value^a^** |
| --- | --- | --- | --- | --- | --- |
| **Proportion of days without heartburn, %** | **n** | **Median (Q1, Q3)** | **n** | **Median (Q1, Q3)** |  |
| ***Response to treatment at Week 2*** |  |  |  |  |  |
| Response according to criterion 1^b^ |  |  |  |  |  |
| Improved | 83 | 92.00 (78.60, 96.40) | 99 | 96.00 (82.10, 100.00) | 0.0478 |
| Not improved | 162 | 50.95 (25.00, 64.30) | 136 | 48.05 (23.70, 65.50) | 0.8963 |
| Response according to criterion 2^c^ |  |  |  |  |  |
| Improved | 168 | 71.40 (57.10, 92.75) | 174 | 81.15 (59.30, 96.40) | 0.0120 |
| Not improved | 77 | 39.30 (4.00, 57.10) | 64 | 24.15 (0.00, 46.55) | 0.0871 |
| ***Baseline NERD grade*** |  |  |  |  |  |
| Grade N | 82 | 64.15 (41.40, 85.70) | 81 | 76.70 (51.90, 92.90) | 0.1490 |
| Grade M | 163 | 60.70 (39.30, 82.10) | 157 | 67.90 (38.50, 92.60) | 0.2146 |
| ***Combination of baseline NERD grade and response to treatment at Week 2*** |  |  |  |  |  |
| Response according to criterion 1^b^ |  |  |  |  |  |
| Grade N and improved | 28 | 93.00 (83.95, 100.00) | 39 | 92.90 (78.60, 96.40) | 0.6631 |
| Grade N and not improved | 54 | 53.60 (28.60, 64.30) | 41 | 53.60 (32.10, 71.40) | 0.5627 |
| Grade M and improved | 55 | 89.30 (77.80, 96.40) | 60 | 96.40 (82.10, 100.00) | 0.0042 |
| Grade M and not improved | 108 | 49.05 (25.00, 64.15) | 95 | 44.40 (21.40, 64.30) | 0.6452 |
| Response according to criterion 2^c^ |  |  |  |  |  |
| Grade N and improved | 59 | 69.00 (46.70, 92.90) | 61 | 79.30 (64.30, 96.00) | 0.0376 |
| Grade N and not improved | 23 | 50.00 (13.80, 65.40) | 20 | 22.95 (5.55, 51.80) | 0.1600 |
| Grade M and improved | 109 | 73.30 (59.30, 90.30) | 113 | 82.10 (59.30, 96.40) | 0.1032 |
| Grade M and not improved | 54 | 33.90 (0.00, 56.70) | 44 | 25.00 (0.00, 46.40) | 0.2613 |

M, minimal changes to the mucosa; N, normal mucosa; NERD, nonerosive esophageal reflux disease; Q1, quartile 1; Q3, quartile 3.

^a^Wilcoxon rank-sum test for difference in median between vonoprazan and placebo.

^b^Criterion 1: Improved, the patient experienced heartburn <2 days of the 7 days prior to Week 2 (Day 8 through Day 14); Not improved, the patient experienced heartburn ≥2 days of the 7 days prior to Week 2.

^c^Criterion 2: Improved, proportion of days the patient experienced heartburn during the treatment period up to Week 2 (Day 14) was lower than that during the run-in period; Not improved: the proportion of days the patient experienced heartburn during the treatment period up to Week 2 (Day 14) was equal to or larger than that during the run-in period.
